# Using Openly Accessible Resources to Strengthen Causal Inference in Epigenetic Epidemiology of Neurodevelopment and Mental Health

**DOI:** 10.3390/genes10030193

**Published:** 2019-03-01

**Authors:** Esther Walton, Caroline L. Relton, Doretta Caramaschi

**Affiliations:** 1 Medical Research Council Integrative Epidemiology Unit, University of Bristol, Bristol BS8 2BN, UK; caroline.relton@bristol.ac.uk (C.L.R.); d.caramaschi@bristol.ac.uk (D.C.); 2 Population Health Sciences, Bristol Medical School, University of Bristol, Bristol BS8 2BN, UK; 3 Department of Psychology, University of Bath, Bath BA2 7AY, UK

**Keywords:** DNA methylation, epigenetics, mental health, neurodevelopment, causal inference, Mendelian randomization

## Abstract

The recent focus on the role of epigenetic mechanisms in mental health has led to several studies examining the association of epigenetic processes with psychiatric conditions and neurodevelopmental traits. Some studies suggest that epigenetic changes might be causal in the development of the psychiatric condition under investigation. However, other scenarios are possible, e.g., statistical confounding or reverse causation, making it particularly challenging to derive conclusions on causality. In the present review, we examine the evidence from human population studies for a possible role of epigenetic mechanisms in neurodevelopment and mental health and discuss methodological approaches on how to strengthen causal inference, including the need for replication, (quasi-)experimental approaches and Mendelian randomization. We signpost openly accessible resources (e.g., “MR-Base” “EWAS catalog” as well as tissue-specific methylation and gene expression databases) to aid the application of these approaches.

## 1. Epidemiological Evidence Linking Epigenetics and Mental Health

Mental health and neurodevelopmental disorders are under the influence of both genetic and environmental factors. Epigenetic mechanisms regulate gene expression and are potential mediators of both these genetic and environmental effects on mental traits and disorders. Of the known epigenetic processes involved in gene regulation, DNA methylation, which consists of the covalent addition of a methyl group to a cytosine base at CpG dinucleotides, is the most widely studied. The main reason for its popularity is the availability of cost-effective, high throughput laboratory assays that utilise DNA extracted using standard protocols. To date, most epigenetic studies of mental health have measured DNA methylation at the genome-wide level using Illumina Infinium 450K or EPIC arrays in peripheral blood or saliva samples, since these tissues are most commonly available in large studies.

Epidemiological studies that have investigated the association of DNA methylation with mental health traits and conditions in peripheral blood or saliva using the Illumina 450K arrays were identified in a semi-systemic manner by searching within PubMed. The characteristics of the studies are summarised in Table 1. While this search is not meant as a systematic review, it provides examples of studies that investigated the link between DNA methylation and brain-related processes in peripheral tissues. Associations of DNA methylation variation measured in peripheral blood in relation to schizophrenia are among the most widely published so far. In the largest study to date, a comparison of 689 men affected by the disease and 645 controls reported over 900 methylation-variable sites across the genome. Although the authors applied a more relaxed threshold (false discovery rate (FDR) *p* < 0.2) in the discovery sample, many methylation sites were replicated in an independent sample with effects consistent in size and direction [1]. Other associations have been reported, linking methylation-variable loci with suicidal behaviour within individuals with bipolar disorder [2], for depressive symptoms within the elderly [3], self-reported wellbeing [4] and panic disorder in adulthood [5]. However, in some instances, conflicting evidence can be found [6] or only very weak evidence is provided, as seen in a study on post-traumatic stress and major depressive disorder [7]. With respect to neurodevelopment, DNA methylation differences were reported in relation to educational attainment and cognitive abilities measured in adulthood [8], attention-deficit hyperactivity disorder [9], oppositional defiant disorder [10], multiple risk behaviours [11], substance abuse [12], early-onset conduct disorder [13] and childhood physical aggression [14], with weaker evidence for an association with violent aggression and diagnosed autism spectrum disorders [15,16,17]. Neurological conditions that showed differences in blood-based DNA methylation when compared to controls include mesial temporal lobe epilepsy [18], narcolepsy [19] and Parkinson’s disease [20].

## 2. Challenges to Assess Causality

Although there are indications that peripheral DNA methylation could be a plausible mechanism that leads to certain brain-related conditions, causality is often difficult to establish in epigenetic epidemiology. Many studies based on epigenome-wide associations are observational and do not allow for a direct assessment of whether the observed DNA methylation differences are a cause, consequence or confounder for the disease of interest. 

Firstly, evidence is often based on studies with small sample sizes without replication. Even if the effects are replicated across studies, they might arise due to similar confounding structures in the data sets, such as the distribution of tobacco smoking behaviours. Even after adjusting for self-reported smoking, residual confounding could still be present due to reporting bias. For example, the association study of DNA methylation on educational attainment has revealed that all sites linked with education have previously been associated with smoking behaviour. Since smoking is often negatively correlated with years of education, this suggests that the observed association between DNA methylation and education is largely due to confounding, rather than describing a causal relationship [21]. 

Another possible scenario where DNA methylation changes are not causal for a disease arises when the disease manifestation itself causes changes in DNA methylation, also referred to as reverse causation. This could arise in cross-sectional studies, where the samples for DNA methylation analysis are obtained at the same time point as the administration of a questionnaire to assess the outcome of interest, or where the methylation measurement was taken after the diagnosis of a disease was made. For instance, in the large epigenome-wide association study (EWAS) on major depressive disorder, DNA methylation was measured after the diagnosis was made. Hence, based on the association study alone, it is impossible to disentangle whether epigenetic changes are the cause or consequence of the disease [3].

Most human epigenetic studies of mental health are based on peripheral samples. Although in some cases methylation changes occur in CpG sites linked to genes that have relevant brain functions, it is often challenging to relate changes in peripheral methylation to the development of a condition that affects the central nervous system (CNS). This problem is of relevance mainly because DNA methylation in the brain of living individuals cannot be quantified. Post-mortem samples, while rare, only allow the assessment of DNA methylation changes after the disease has manifested [23], as, for instance, in an EWAS of autism spectrum disorder conducted across several brain regions [24]. In this case, epigenetic changes could be confounded by treatment effects, as DNA methylation changes have been reported, for instance, in relation to antipsychotic treatment [25]. 

The ‘gold standard’ experimental approach used to seek causal evidence is the randomised controlled trial (RCT). However, this is not a feasible option for DNA methylation research, as it is not yet possible or ethical to undertake an RCT with DNA methylation as the primary controlled exposure. Some studies have taken advantage of RCTs set up with other primary exposures and subsequently measured DNA methylation as a surrogate or intermediate, but these have tended to be serendipitous, relying on RCTs that have collected DNA samples for other purposes (see below for further discussion of this issue).

Animal studies, particularly in the laboratory, have the advantage of allowing for controlled experimental conditions and access to specific tissues other than peripheral blood, therefore avoiding the issue of confounding and the otherwise limited inferences that can be made with respect to tissue specificity. In mouse studies, DNA methylation can, for example, be manipulated by deleting the genes coding for DNA methyltransferases (*Dnmt1/Dnmt3a/Dnmt3b*), the enzymes that catalyse the transfer of a methyl group to a cytosine nucleotide. A study by Hutnick et al. [26] showed that the deletion of *Dnmt1*, even when restricted to the forebrain, caused widespread hypomethylation, neuronal degeneration and behavioural impairment in learning and memory. This is in line with other mouse studies, where *Dnmt1* deletion seemed to cause increases in anxiety-like behaviour and deleting both *Dnmt1* and *Dnmt3* led to synaptic abnormalities with functional consequences for hippocampal plasticity [27,28]. These studies indicate a causal link between overall DNA methylation and brain-related traits, however they do not allow for the identification of specific methylation loci within the genome at which the changes in DNA methylation might be exerting their influence. Recently, with the technology of the CRISPR-Cas9 system applied in vivo to laboratory mice, it has become possible to demonstrate that DNA methylation at the *FMR1* gene causes the molecular and physiological phenotype of fragile-X syndrome [29]. While fragile-X syndrome has a specific and detectable molecular phenotype (lack of FMR1 protein), the limitation of most animal studies is that many human psychiatric diseases are defined by behavioural traits that can only be partially observed in other species. Most animal models are based on the resemblance of the behavioural symptoms and therefore mostly correspond to a sub-set of symptoms and traits of the modelled human psychiatric diseases, rather than the full disease. Similarly, the pathological mechanisms leading to the human psychiatric conditions might not necessarily correspond to the changes observed in the animal models that only partially mimic the human condition.

## 3. Epidemiological Approaches to Investigate Causality

### 3.1. Strength and Robustness of the Associations

True epigenetic associations often tend to replicate in population samples with similar characteristics and confounding structures, thus the associations observed could be due to real effects or to other non-causal explanations. To assess the strength and robustness of the associations it is recommended, where feasible, to work collaboratively across multiple studies, as true causal associations ought to be reproduced across studies with different confounding structures. Such collaborations can be achieved within consortia, where several studies with available epigenomic data can contribute to addressing the same research questions according to agreed and standardized analysis plans. Selected examples of such consortia that have been used in the field of epigenetic epidemiology are listed in Table 2 below.

For these cross-cohort analyses, it is, however, essential to standardize pre-processing steps, including normalisation, quality checks, and epigenome-wide association study (EWAS) analyses procedures. Data sharing is often a limiting factor in analyses of this type and harmonizing data across studies can sometimes be resource intensive. Software packages have been developed to facilitate such analyses. For example, the *meffil* R package, which was created to enable cross-cohort harmonization without data sharing, is available for download at https://github.com/perishky/meffil [33]. 

Where there is no opportunity for collaboration, or the phenotypes of interest are not available in consortia, it is sometimes possible to access DNA methylation data and their association with the phenotype from openly available online repositories, such as Gene Expression Omnibus (GEO) (https://www.ncbi.nlm.nih.gov/geo/). In the GEO repository, data can be downloaded or analysed online with the interactive GEO2R tool [34]. 

Replicating associations across different datasets also provides an opportunity to verify that results are not due to technical artefacts. Although replication does not necessarily increase the likelihood of associations being causal, it can be a further step in supporting the veracity of the observed association. For instance, investigating the same CpG sites-trait associations across the Illumina 450K or the more recent EPIC array or using different techniques, including pyrosequencing, bisulphite sequencing and qPCR, will strengthen the inferences that can be made with respect to the confidence in true associations.

### 3.2. Experimental and Quasi-Experimental Approaches

The conventional epidemiological design to investigate causality, an RCT requires the participants to be randomly assigned to groups that are similar except for the exposure of interest (here, DNA methylation). Although theoretically it is possible to conduct an RCT of a demethylating agent and assess its impact on a mental health outcome, a targeted manipulation of specific methylation sites is currently not achievable with the available tools. 

RCTs are, however, more tractable where methylation is considered as a secondary outcome to investigate the effects of an intervention. For example, RCT designs were exploited to assess the effects of pollution [35] and folate intake [36] on DNA methylation. Linking changes in methylation, which have been identified to be a causal consequence of environmental exposures, to psychiatric disorders could be an interesting and worthwhile extension of such findings.

Natural experiments, where populations are exposed to an unplanned disaster or event, provide valuable data to reveal changes in DNA methylation that are causal for psychiatric conditions. For example, methylation changes due to prenatal exposure to the Dutch famine [37] have been shown to cause changes in mental health in adulthood [38] and suggest that DNA methylation could be a potential mediating mechanism. Similarly, prenatal maternal stress due to a significant ice storm in Quebec in 1998 affected DNA methylation [39] and autism-related traits [40].

### 3.3. Mendelian Randomization

One widely adopted approach to strengthen causal inference is the method of Mendelian randomization (MR), a form of instrumental variable analysis. In MR, the instrument is comprised of one or more genetic variants that are robustly associated with the exposure of interest. As individuals inherit alleles at random, these individuals are assigned to experience a higher-than-average dosage of the exposure. 

Mendelian randomization relies on the availability of genetic variants to use as instrumental variables (for a discussion on additional assumptions, see [41,42]). Where genetic variants can be identified that correlate strongly with DNA methylation levels, MR can be applied to study causal effects of DNA methylation on mental health. Depending on the research question, the sample characteristics and data availability, different MR methodologies can be applied, such as one-sample, two-sample, bidirectional, multivariable and two-step MR, the details of which can be found elsewhere [43,44]. Due to limitations in data availability and the computational resources required, MR has predominantly been performed to date on selected methylation loci (e.g., top hits of a robust EWAS), with a few notable exceptions [45,46]. However, with the advent of more detailed data on genetic variants that tag methylation variation, the approach promises to be more widely adopted.

#### 3.3.1. Instruments for Epigenetic Mendelian Randomization Analysis 

Potential instruments for DNA methylation are single nucleotide polymorphisms (SNPs) that are strongly associated with methylation at the CpG sites of interest—often referred to as methylation quantitative trait loci (mQTL). These can be found in online databases that have performed genome-wide association studies (GWAS) of DNA methylation (Table 3). The overwhelming majority of catalogued mQTLs have been derived from populations of European ancestry and are based on peripheral blood DNA, raising the issue of whether the same SNP–DNA methylation relationship is observed in other ethnicities or tissues. Emerging evidence suggests that this assumption might be plausible in some instances [47]. However, as DNA methylation is often tissue-specific, brain-tissue specific databases (Table 3) can be used to identify mQTLs when the hypothesis implies a biological mechanism that acts via changes in brain DNA methylation.

Alternatively, blood-derived mQTLs can be used in MR when an EWAS of a brain-related trait has been conducted in blood and it is plausible that changes in methylation in blood cells are reflected in changes in brain activity, for instance, via circulating hormones that cross the blood–brain barrier (see Section 3.4.1 for a more detailed discussion). 

Some of the resources listed in Table 3 are based on data from specific developmental periods (e.g., foetal sample, cord blood), however, our ability to use these resources in a developmentally sensitive manner is still restricted and heterogeneity in ethnicity and cell type composition between the target and the reference datasets limits any conclusions drawn from these analyses.

Most mQTLs are cis-associations, i.e., they are located proximal to the CpG of interest. Cis-SNPs have large effects on the CpGs in their proximity, whereas trans-SNPs have smaller effects and tend to act polygenically on several target loci. For these reasons, cis-SNPs, rather than trans, are preferred as instruments for use in MR.

#### 3.3.2. Methodologies in Epigenetic Mendelian Randomization Analyses

**Figure 1:**
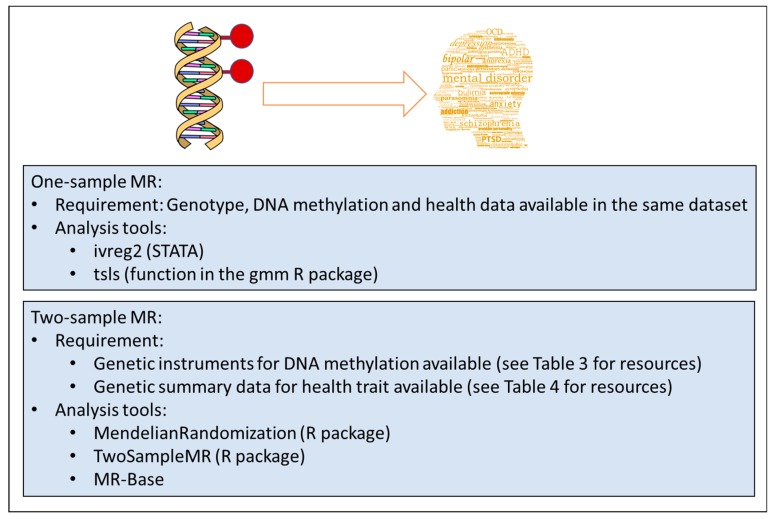
Overview of methodologies in epigenetic Mendelian Randomization (MR) analyses.

If mQTLs are available for the CpGs of interest, they can be used as instruments for MR. In studies where genotypes, DNA methylation data and the outcome (e.g., a mental health trait), are available, it is possible to perform one-sample MR using the 2-stage-least-square regression (Figure 1, top panel). This is easily implemented with the *ivreg2* command in the STATA software or the function *tsls* in the *gmm* R package (https://cran.r-project.org/web/packages/gmm/index.html) [51].

When this data is not available, a two-sample MR approach can be used (Figure 1, bottom panel). This relies on extracting the genotype-methylation (G-M) summary statistics (beta regression coefficients and standard errors) from one study and the genotype-outcome (G-O) statistics from another, independent study. For one SNP, the causal estimate is the ratio of the genotype-outcome beta coefficient divided by the genotype-methylation beta coefficient. The standard error of the causal estimate is estimated via the delta-method as described in Thomas et al. [52]. When at least three genetic variants are available, the G-M/G-O ratio estimates are meta-analysed using standard meta-analysis methods, such as the inverse variance weighted approach with fixed or random effects models. Two-sample MR can be easily performed using the MR-Base online tool (http://www.mrbase.org/) and the *TwoSampleMR* R package available for download at the github online repository (https://github.com/MRCIEU/TwoSampleMR) [53]. Similarly, the *MendelianRandomization* R package performs two-sample MR using existing summary data on genetic associations with exposure and outcome [54]. When several SNPs are available it is useful to choose the MR-Egger model, which provides a test for horizontal pleiotropy and a pleiotropy-adjusted causal estimate [55]. However, this method has lower power and is recommended primarily as a sensitivity analysis. GWAS summary statistics for the G-O associations can be found in several online databases (Table 4).

Following this strategy, two-sample MR has recently been applied to test for a causal effect of methylation in the *DRD4* gene on physical aggression and did not support a causative link [14].

The direction of the association, if not known a priori, can be queried using bi-directional MR, where both a causal effect of methylation on the trait and a causal effect of the trait on methylation are estimated. Effectively, this procedure involves two MR analyses, requires a set of independent SNPs for each analysis and can be carried out within the one-sample or the two-sample setting. 

When the research interest is to estimate the effect of an exposure on an outcome via DNA methylation, to supplement the conventional observational mediation approach, it is useful to adopt an MR strategy that involves two MRs, one from exposure to methylation and one from methylation to the outcome of interest. In the two-step MR approach, the SNPs used as instruments for each step need to be independent. Each MR step adopts the usual assumptions for MR and is performed using the same general principles and methods for MR. This implies that several independent study samples are needed to obtain the summary statistics for the genotype-exposure (G-E), G-M and G-O associations, which can be identified using the resources listed in Table 3 and Table 4. Two-step MR has been applied to test the causal role of prenatal nutrients involved in the one-carbon metabolism on schizophrenia via epigenetic changes [60] and to reveal DNA methylation as a mediator between the exposure to prenatal vitamin B_12_ and cognitive abilities [61]. 

Other methods using genetic variants to strengthen causal inference are based on the integration of genome-wide genetic and epigenetic data with the disease of interest, using polygenic risk scores (PRS) for the disease and co-localisation analyses. PRS are defined as the sum of trait-associated alleles across many genetic loci, weighted by the GWAS effect size. Similar to the MR approach, the epigenetic and phenotypic variation associated with PRS is less likely to be confounded by lifestyle exposures such as smoking and environmental factors such as pollution and is less prone to reverse causation. For example, EWAS studies on schizophrenia where PRS rather than diagnosis were used in the analysis have identified DNA methylation differences at novel CpGs [62]. Furthermore, Bayesian co-localisation analysis, where the results of a GWAS of methylation at the CpG sites and the results from an independent GWAS for schizophrenia were compared, supported the hypothesis that some of the genetic variants within the overlapping sites had a regulatory role in the disease via influencing DNA methylation [63]. PRS for brain-related disease can be computed using summary statistics from published GWAS (see Table 4 for a list of resources; to derive polygenic scores, see https://choishingwan.github.io/PRSice (version 2.1.9), https://www.cog-genomics.org/plink/1.9/score (version 1.9) and [64]). Bayesian colocalization analysis can be performed using existing summary data from mQTL databases and the *coloc* R package (https://cran.r-project.org/web/packages/coloc/) [65]. 

### 3.4. Plausibility of Biological Mechanisms

#### 3.4.1. A Word of Caution: Mechanism vs. Biomarker

The excitement of obtaining an epigenetic signal that is strong, robust and potentially causal can be exhilarating. However, before deriving conclusions about the ‘aetiological mechanism of disease’, it is advisable to recall the original aim of the study. Frequently, the aim is to identify causes of disease, which is imperative for interventions to be successful. On the other hand, establishing non-causal associations (often referred to as biomarkers, see below) can be useful in prediction. However, a biomarker can be causal or non-causal. Whether the aim is to identify a causal pathway and/or a biomarker (of risk or of disease) should be set out in the initial stages of the project. Caution is advised with respect to the conclusions that can be drawn from the study design and data in terms of biological mechanisms. The interpretation of results will differ, depending on the underlying assumptions about the likelihood of system-wide effects of the exposure (i.e., genetic or environmental causes of disease), the relationship between the studied tissue and the primary tissue of pathophysiology. In most cases, methylation profiles would have been obtained from peripheral tissues (blood or saliva), with a small proportion of studies using post-mortem brain tissue. 

**Figure 2:**
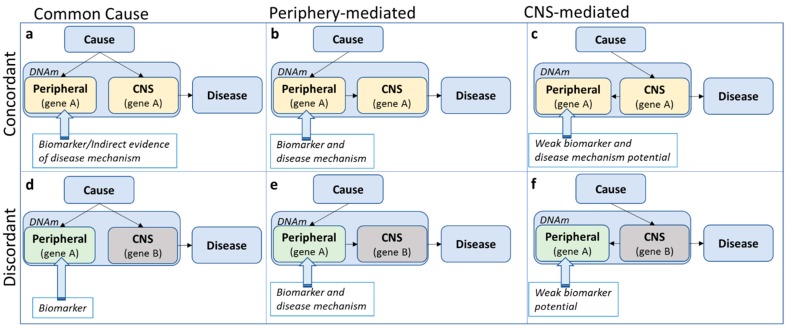
Three potential scenarios (by column) describing the relationship between peripheral and central nervous system (CNS) DNA methylation (DNAm) profiles within the pathway from cause to disease, assuming either consistency in effect of the risk exposure on DNA methylation across tissue (i.e., concordance; top panel) or discordance (bottom panel).

Under the assumption that the causal (but not necessarily initial, see argument below) tissue of pathophysiology is the brain, at least three potential scenarios are possible to describe the relationship between peripheral and CNS methylation profiles: A shared common cause, periphery-mediated or CNS-mediated pathways to disease (left, middle and right panels in Figure 2). Note that a scenario in which DNA methylation is a direct consequence, rather than a precursor, of disease, is an equally likely possibility, but not the focus of the current discussion. A mechanistic interpretation of findings based on peripheral tissue only makes sense assuming that the initial cause of pathophysiology originates in the periphery (Figure 2b,e) or at the very least assuming concordance of methylation patterns across tissues (top panel Figure 2, although see below for additional assumptions).

**Figure 3:**
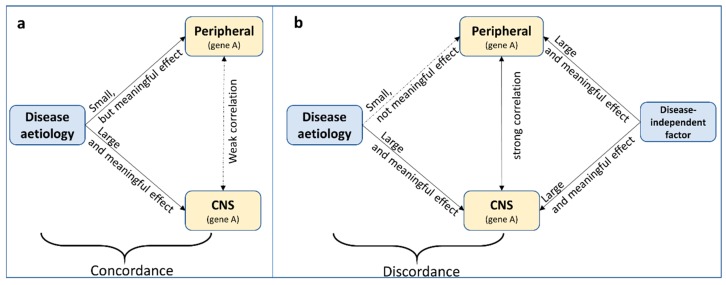
Two scenarios demonstrating that correlation of DNA methylation profiles across tissues is neither (**a**) necessary nor (**b**) sufficient for cross-tissue concordant effects.

‘Concordance’ in this case shall be defined as the consistency in effect of the exposure (i.e., the cause of disease) on DNA methylation across tissue. This is different from ‘correlation’ of DNA methylation across tissue. For example, *relative* (but meaningful) perturbations in DNA methylation due to an exposure might be comparable across tissue, while *absolute* DNA methylation levels themselves are less correlated across tissues (Figure 3a). This assumes that small levels of perturbations can have large effects in some but not in other tissues. Likewise, without knowing what precisely causes cross-tissue correlations in DNA methylation, DNA methylation levels might be correlated across tissue, but the effect of an exposure on DNA methylation in each tissue is different (Figure 3b). Therefore, while correlation of DNA methylation profiles across tissues is often an important indication, it is neither necessary nor sufficient for cross-tissue concordant effects.

All too often, cross-tissue concordance and correlation are implicitly assumed and findings are interpreted as potentially mechanistic. However, there is evidence that cross-tissue *correlation* seems to be the exception, rather than the norm [66]. *Concordance* of methylation profiles across tissues is hardly ever investigated, due to the difficulty (and cost) in measuring the effect of a risk factor on DNA methylation across several tissues in the same individuals. The notable exception of this is the investigation of tissue-specific mQTLs. For online available resources to investigate cross-tissue concordance and correlation, see Section 3.4.2 and Section 3.4.3.

Even in the case of cross-tissue concordance, it is easy to overstate risk pathways to disease. In the concordant, common cause scenario (Figure 2a), the tendency is to assume system-wide causal effects, but it might be equally likely that a disease risk factor impacts methylation of the same gene in different tissues independently. In all concordant scenarios (Figure 2a–c), concordant gene function across tissues is presumed, although genes can have different functions in different tissues. For example, assuming that in an analysis based on data from whole blood, a methylation site was identified with a potential relevance for serotonin function. In the periphery, the primary function of serotonin is digestion, while in the CNS, serotonin is mainly involved in sleep and mood [67]. In the ‘shared common cause’ scenario (Figure 2a), we do not need to focus on digestion-related functions, as these are not likely to be involved in the disease pathophysiology. In the ‘periphery-mediated’ scenario (Figure 2b), however, digestion should be a main pathway-of-risk, while in the ‘CNS-mediated’ scenario (Figure 2c), digestion is, if anything, a downstream pathway of disease. Any mechanistic interpretation of findings depends fundamentally on which scenario is most likely.

When concordance is not assumed (Figure 2d–f), the default position is often that, even though the epigenetic variation is not likely to be mechanistically involved, it may act as a biomarker of disease risk. However, the precise ‘biomarker’ definition referred to is often not clear. According to the National Institute of Health Biomarkers Definition Working group, a biomarker is ‘a characteristic that is objectively measured and evaluated as an indicator of normal biologic processes, pathologic processes or biological responses to a therapeutic intervention’ [68]. While it is beyond the scope of this review to discuss the role of DNA methylation as a biomarker of risk or disease, this term should not be used too lightly. Biomarkers should be easily (in terms of tissue accessibility) and robustly measurable with little measurement error, reproducible across studies (e.g., it is not advised to claim biomarker potential based on a single study without replication) and have predictive power (or alternative advantages, such as reducing costs). Finally, it should be clear what exactly the established biomarker indexes (risk, disease or treatment). While it is often claimed that methylation-based biomarkers have the potential to inform intervention strategies, studies designed to explicitly demonstrate this are rarely seen [69]. 

It is impossible to test these scenarios (Figure 2) directly without access to longitudinal and repeated measures of both peripheral and brain tissue in living humans, but their likelihood can be assessed by using tissue-specific causal inference method such as Mendelian randomization (see Section 3.3) and the increasing body of online resources as described in the following sections.

#### 3.4.2. Biological Characterisation

Characterising the biological relevance of an identified methylation site is often part of an epigenome-wide analysis, regardless of whether a potential disease mechanism has been established. While methylation sites are often primarily viewed in relation to the nearest coding gene, it can be equally important to consider DNA methylation in the context of regulation of gene expression via impacting chromatin accessibility and transcription factor binding. For instance, studies have confirmed that DNA methylation around the transcription start site is largely associated with reduced gene expression locally [49]. In a study based on brain samples, DNA methylation and histone modifications were located in regulatory regions and seemed to mediate the association of genetic variants with gene expression [70]. Many of those epigenomic loci were also replicated in peripheral blood samples and were associated with psychiatric diseases, such as schizophrenia and bipolar disorder. To characterize the biological context of a methylation site, the results of an EWAS can first be matched to the annotation file usually provided with the data, or openly accessible online (Illumina 450k and EPIC array annotation are, for example, available via various R packages such as *meffil* [33]). This will provide CpG information on genomic location, SNPs located in or close to the probe, associated genes and location with respect to the transcription start site of these genes or CpG islands. Furthermore, information is provided on low- or high-CpG density regions associated with Functional Annotation of the Mouse/Mammalian Genome (FANTOM) 4 promoters [71], although the reader should keep in mind that this information was based on human myeloid leukaemia cell lines and is not specific to CNS tissue. Finally, in the annotation file the reader will find information on enhancer elements, DNase I Hypersensitivity Sites, open chromatin regions and transcription factor binding sites (all based on the Encyclopaedia of DNA Elements (ENCODE) data [72]).

Whenever possible, however, querying several databases (see Table 5 for selected resources) is advocated to corroborate results and to summarize all findings to avoid selective reporting. Also, to achieve a more meaningful interpretation of the regulatory nature of the genomic region in question, investigating these regulatory characteristics in a cell-type specific manner is advisable, which can be achieved using ENCODE data (www.encodeproject.org), usually via platforms such as genome.ucsc.edu. For example, DNase I hypersensitivity clusters—indicative of regulatory chromatin regions that are sensitive to cutting by the enzyme DNase—can be viewed for 125 cell types (including cells derived from blood and brain tissue) as part of the ENCODE project. Histone marks and transcription levels are available for up to nine cell lines (including blood, embryonic stem cells and skeletal muscle, among others). Transcription factor binding sites are listed for 161 factors in 91 cell types (for a list on cell types, see here: https://genome.ucsc.edu/cgi-bin/hgEncodeVocab?type=%22cell%22). Note that information on CNS-specific cell types is not always available but high (or low) correspondence across these diverse cell types could indicate similarly (un-)correlated profiles in brain tissue. For cell-type specific profiles related to brain tissue, a suggestion could be to investigate DNase I and histone mark data from the Roadmap Epigenetics Project (http://www.roadmapepigenomics.org/data/) that assayed ten different brain regions (including the hippocampus, cerebellum and mid-frontal lobe, among others). Note though that DNase I data is only available for foetal brain (not region-specific) and spinal cord tissue. Also note that, to view Roadmap data in the UCSC genome browser, the reader will need to import these tracks via the UCSC Track data hub (https://genome.ucsc.edu/cgi-bin/hgHubConnect) or via http://www.roadmapepigenomics.org/data/. PsychENCODE is a comprehensive resource with exceptional relevance to brain related traits [73,74,75,76,77,78,79,80,81,82,83]. It provides raw and derived transcriptomic, epigenomic, and genomic data of post-mortem adult and developing human brains, both at the single-cell and tissue level. This dataset also includes measures on (hydroxy-)methylation, is based on up to 2000 individuals and incorporates resources such as GTEx, ENCODE and Roadmap Epigenetics Project, discussed above and elsewhere in this article. Data and results can be downloaded from The PsychENCODE knowledge portal (http://www.synapse.org/pec) and from http://resource.psychencode.org/.

After investigating the regulatory nature of the genomic region, it can also be helpful to query whether the CpG itself or the differentially methylated region (DMR) has been implicated in other epigenome-wide analyses, which can be done using a manually curated EWAS catalogue hosted at http://www.ewascatalog.org/. 

Finally, it is advised to investigate: (1) Ehether a CpG-of-interest is under genetic control by identifying potential mQTLs, ideally in a tissue-specific manner (see Section 3.3.1 and Table 3 above for a list of resources); (2) whether a genomic region might show epigenetic supersimilarity, i.e., where the similarity in DNA methylation between twins is greater than expected based on shared genetics, as reported by Van Baak et al. [85]; and (3) whether a CpG-linked gene might be imprinted, meaning that the expression of this gene depends on the parental origin. For a list of imprinted genes, see http://www.geneimprint.com/site/genes-by-species.

#### 3.4.3. Cross-Tissue Comparisons

Cross-tissue correlation (see Section 3.4.1) is an important, but not essential, requirement, even for a mechanistic interpretation of findings (e.g., Figure 2e). In practice, correspondence can be investigated using cell-type specific data on regulatory regions (see Section 3.4.2 and Table 5) and several other openly accessible online resources (Table 6). BECon [86] (https://redgar598.shinyapps.io/BECon/) is based on paired blood and post-mortem brain tissue data from 16 individuals. The user can enter a CpG or gene name to visualize cross-tissue correlation across blood and three brain regions (BA10 (frontal), BA20 (temporal) and BA7 (parietal)). Another online resource with similar functionality is available via https://epigenetics.essex.ac.uk/bloodbrain/, based on matched blood and four post-mortem brain tissues (cerebellum, entorhinal cortex, frontal cortex and superior temporal gyrus) in 74 individuals. These two resources are based on the Illumina 450k array. Methylation data based on bisulphite sequencing are available via MethBase [87] (http://smithlabresearch.org/software/methbase/) and can be imported via the Track hub option (see Section 3.4.2) into the UCSC genome browser. This resource provides information on methylation levels at individual sites, allele-specific methylation and hypomethylated or hypermethylated regions. Furthermore, MethBase does not only allow for comparisons across cell types (frontal cortex, neural progenitor cells, embryonic stem cells and blood tissue cells in humans), but also across development (from 35 days to 64 years in the case of brain tissue data) and across species (including human, mouse, chimp, dog, zebrafish and plants). 

Alternatively, it is possible to test for a tissue-specific enrichment of EWAS probe sets, an option which is currently implemented in eFORGE (http://eforge.cs.ucl.ac.uk/). Relying on data from ENCODE and the Epigenomics Roadmap, eFORGE compares DNase I hypersensitivity site hotspot overlap between an EWAS input list and background probes in a cell-type specific manner.

An alternative technique to investigate cross-tissue correspondence was applied in Linnér et al. [21] using data from the Epigenomic Roadmap Consortium (see Section 3.4.2; although alternative resources such as PsychENCODE listed in Table 5 could also be used). There, the authors calculated average cross-tissue methylation for a selected number of CpG sites linked to educational attainment and derived deviation from this average for a range of tissues (including brain tissue). These tissue-specific measures of deviation were then correlated with EWAS test statistics (z-scores). The authors argued that a lack of correlation between EWAS z-scores of educational attainment and tissue-specific derivation (especially in brain tissue, assumed to be the target tissue of interest) indicated an absence of brain-tissue specific effects and might be suggestive of confounding. Of note, this method is based on average methylation levels across tissue and not on correlations (i.e., methylation profiles might be correlated across tissues, but at different absolute methylation levels).

Finally, there is some evidence that the effects of mQTLs on methylation can be stable across tissues [48], although large-scale investigations across a wide range of tissue types (including brain tissue) are still missing. With this in mind, investigating consistency of mQTL effects across tissues (using resources described in Section 3.3.1) can be helpful to obtain some indirect evidence for or against cross-tissue concordance. 

#### 3.4.4. Tissue-Specific Gene and Protein Expression 

It is generally assumed that DNA methylation influences gene expression. However, this issue is still extensively debated [89] and the absence of a functional effect of methylation of gene expression does not preclude the possibility of a meaningful, causal mechanism. Still, it can be highly informative to investigate whether a gene linked to variation in DNA methylation at a site-of-interest also shows variation in its level of expression in the tissue-of-interest. The following section and Table 7 provide an overview of online resources to assess gene expression profiles by tissue and across development.

The Human Protein Atlas (https://www.proteinatlas.org/humanproteome) is an excellent resource to investigate in which tissues a gene-of-interest is expressed in absolute terms, and also whether the expression of such a gene is elevated in the target tissue relative to average expression levels in all tissues. Lists of whole groups of genes that are preferentially expressed in certain tissues (e.g., n = 1460 genes are listed to show elevated expression profiles in brain tissue relative to all other tissues) can be used to test for enrichment of brain-expressed genes in EWAS results.

The Genotype-Tissue Expression project (GTEx, https://gtexportal.org/home/) provides similar options, listing information on tissue-specific gene expression, regulation and expression quantitative trait loci (eQTL) information. Importantly, the eQTL function allows users to investigate tissue-specific eQTL effects (for example of SNPs that have already been identified to be mQTLs). 

To gain insight into gene expression profiles across development, the reader is encouraged to consult the EMBL-EBI expression atlas (https://www.ebi.ac.uk/gxa/home), which displays data from a range of resources (including NIH Epigenomics Roadmap, ENCODE and GTEx). 

Several resources are of particular relevance to brain tissue-specific gene expression. The Allen Brain Map portal (http://portal.brain-map.org/) provides a range of useful data, including the Human Brain Atlas and the Developing Human Brain resources. The former is a unique multimodal atlas of the human brain, integrating highly detailed anatomic and genomic information. The user can search for a gene-of-interest and visualize its expression profile in different brain regions using high-resolution, MRI-based 3-D histology scans.

The BrainSpan Atlas of the Developing Human Brain (http://www.brainspan.org) provides information on the human transcriptome (RNA sequencing and exon microarray data) across different brain regions and development. The BrainCloud application informs on genome-wide gene expression and their genetic control in the dorsolateral prefrontal cortex of normal subjects across the lifespan (http://braincloud.jhmi.edu).

The PsychENCODE project combines data from several resources (including GTEx and BrainSpan) to characterize a large spectrum of genomic elements with the human brain, including gene expression as well as multi-QTL maps (for expression, chromatin, transcript expression and cell fraction), enhancers, splice variants and co-expression modules, often specific to cell type, brain region or developmental period. For a more detailed discussion on brain-based resources, see Keil et al [90].

Finally, it is important to note that gene expression levels (either in absolute terms or relative to average levels across tissues) can be misinterpreted. For example, *DRD4* (coding for the dopamine D4 receptor) does not appear to be preferentially expressed in brain tissue, but it would be misleading to come to the conclusion that *DRD4* has no role in psychopathology, as numerous studies have demonstrated DRD4 functioning to be involved in emotion and complex behaviours such as novelty seeking [94,95,96]. Furthermore, there is a renewed interest in dopamine D4 receptor-based pharmacological treatments for substance use and Parkinson’s disease [97]. As highlighted throughout this review, molecular phenotypes including DNA methylation and gene expression vary over time and across tissues, meaning that any measure will be specific to the temporal context at which the sample was taken, thus limiting the inferences that can be made with respect to cause.

#### 3.4.5. Gene Ontology Analysis

At last, it can be of interest to carry out an ontology analysis (or, relatedly, pathway or gene property analyses) to investigate whether the most associated CpG probes cluster within distinct biological functions. A plethora of online resources is available for ontology analyses and the reader is referred to excellent reviews on the topic [98,99]. In general, analysis tools with the option to carry out tissue-specific analyses are recommended. For example, FUMA (http://fuma.ctglab.nl, [100]) tests the relationships between tissue-specific gene expression and disease-gene associations, using gene expression data from GTEx and the BrainSpan project, among others. As this resource was primarily designed for genetic data, the user needs to map CpGs first to a gene before carrying out the analysis using the GENE2FUNC option. With this functionality, Linnér et al. [22] reported that genes closest to CpG probes linked to educational attainment were not preferentially expressed in brain tissue, suggesting that findings might have been driven by confounding factors.

## 4. Strengths and Limitations 

Epigenetic epidemiological studies of mental health and related phenotypes continue to be the focus of much interest with the hope of enhancing understanding of the biological mechanisms underlying the aetiology and progression of psychiatric diseases. However, they still present challenges and limitations. 

The platforms to generate data that have been most widely employed sample only a very small portion of CpG sites in the genome. Studies using sequencing-based approaches, such as a recent methylome-wide association study of major depressive disorder that measured DNA methylation in 28 million CpGs, promise to unlock more information on epigenetic variation and will unravel more insights into the role of methylation in mental health [101]. Moreover, while the majority of the current studies focus on CpG methylation, DNA methylation is also present at non-CpG sites, particularly in brain tissue, suggesting a potential role in neurodevelopment and mental health [102]. Methylome sequencing only recently allowed the characterisation of non-CpG methylation in brain tissue [103] but could provide an additional avenue to discover novel effects in relation to neuropsychiatric traits.

Mendelian randomization is proving to be a useful tool to strengthen causal inference and explore molecular mediation by DNA methylation. It does, however, have recognized limitations and is unlikely to provide definitive evidence of causal pathways without triangulation using complementary approaches in epidemiology and other disciplines.

Epidemiological studies of methylation and brain-related processes using peripheral tissue alone may not be able to unravel true biological mechanisms, but the associations found can be translated in useful biomarkers (whether causal or not) for diseases or their progression and therefore are worth investigating. They can also be used to establish how substantial the contribution of genetic factors to variance in methylation is. Also, it is often of interest to know whether a CpG impacts gene expression (or vice versa), even if not causally linked to disease. Finally, these approaches are useful to explain the correlation between peripheral DNA methylation and brain-based processes, even if these processes index (non-causal) disease correlates. Even with the limitation of not necessarily addressing the issue of causal correlates of psychiatric diseases that could be translated into intervention, peripheral epigenetic associations can answer biological questions that ultimately help the understanding of mental health.

## 5. Future Perspectives and Conclusions

In conclusion, recently developed openly accessible resources allow epigenetic epidemiological studies of mental health and offer multiple opportunities to understand the aetiology and progression of psychiatric conditions. Future advances in software development specific for epigenetics and statistical methodologies for causal inference as well as large biobanks in multiple complementary populations will substantially increase our understanding of mental health and lead to the generation of reproducible results to inform prevention and intervention strategies.

## Figures and Tables

**Table 1:** Epigenome-wide association studies of mental health traits and diseases conducted in peripheral blood. A semi-systematic PubMed search was undertaken (access date 21/11/2018) using the terms ‘DNA methylation’, ‘methylome-wide’, ‘epigenome-wide’, ‘psychiatry’, ‘psychiatric’, ‘behaviour’ and ‘human’. FDR: false discovery rate; DMR: differentially methylated regions.

Trait/Disease	Study Design	Tissue	Sample Size	DNA Methylation Differences	Significance Threshold	Reference
Wellbeing	Population study	Blood	N = 2456	2 CpGs	Bonferroni *p* < 0.05	[4]
Schizophrenia	Case-control	Blood	N = 1339 (discovery); N = 497 (replication)	923 CpGs	FDR *p* < 0.2	[1]
Substance abuse	Population study	Cord blood	N = 244	65 CpGs	FDR q < 0.05	[12]
Suicidal behaviour	Case-control	White blood cells	N = 123	None below threshold	Not specified	[2]
Post-traumatic stress disorder	Clinical study (trauma patients)	Blood	N = 473	None below threshold	FDR *p* < 0.05	[7]
Major depressive disorder	Case-control	Blood	N = 473	None below threshold	FDR *p* < 0.05	[7]
Panic disorder	Case-control	Blood	N = 96	40 CpGs	FDR *p* < 0.05	[5]
Educational attainment	Population study	Blood	N = 10767	9 CpGs	*p* < 1 × 10^−7^	[21]
Mesial temporal lobe epilepsy	Case-control	Blood	N = 60	216 CpGs	*p* < 1.03 × 10^−7^	[18]
Parkinson’s disease	Case-control	Peripheral blood mononuclear cells	N = 38	2 CpGs (identified via multiple methods)	methylation difference >15% and validation with other methods	[20]
Attention-deficit hyperactivity disorder	Population study	Cord blood	N = 828	13 CpGs	FDR q < 0.05	[9]
Oppositional defiant disorder	Population study	Cord blood	N = 671	30 CpGs	FDR q < 0.05	[10]
Depression	Case-control	Blood	N = 200	6 DMRs	Sidak corrected *p* < 0.05	[22]
Cognitive abilities	Population study	Blood	N = 2557–6809	2 CpGs	*p* < 0.05/(420000 CpG x7 traits)	[8]
Depressive symptoms	Case-control	Blood	N = 47	None below threshold		[6]
Depressive symptoms	Population study	Blood	N = 7948 (discovery); N = 3308 (replication)	3 CpGs	*p* < 1.03 × 10^−7^	[3]
Narcolepsy	Case-control	Blood	N = 46	14 CpGs	FDR *p* < 0.05	[19]
Violent aggression	Clinical study (schizophrenia patients)	Peripheral blood mononuclear cells	N = 134 (discovery)	Weak differences	*p* < 1 × 10^−6^	[15]
Physical aggression	Population study	Buccal (discovery); peripheral T cells (replication)	N = 119 (discovery); N = 38 (replication)	4 CpGs; 2 DMRs	FDR q < 0.05	[14]
Early-onset conduct disorder	Case-control	Cord blood	N = 260	7 CpGs	FDR q < 0.05	[13]
Multiple risk behaviours	Population study	Blood	N = 227–575	2 CpGs	FDR q < 0.10	[11]
Autism spectrum disorder	Case-control	Blood	N = 1311	None below threshold	*p* < 1.12 × 10^−7^	[16]
Autism spectrum disorder	Case-control	Cord blood	N = 1263	None below threshold	*p* < 1 × 10^−7^	[17]

**Table 2:** Selection of consortia in the field of epigenetic epidemiology.

Resource	Description	Link
Pregnancy and Childhood Epigenetics (PACE) consortium [30]	Focus on the effect of early life exposures on DNA methylation in childhood	https://www.niehs.nih.gov/research/atniehs/labs/epi/pi/genetics/pace/
Cohorts for Heart and Aging Research in Genomic Epidemiology (CHARGE) [31]	Focus on facilitating genetic and epigenetics meta-analyses and replication opportunities among cohort studies	http://www.chargeconsortium.com/
Genetics of DNA Methylation (GoDMC) consortium [32]	Focus on the genetic basis of DNA methylation variation in participants of different ages and ethnicities	http://www.godmc.org.uk/information.html

**Table 3:** Resources that can be used to identify genetic effects on DNA methylation probes. mQTL: methylation quantitative trait locus.

Resource	Description	Link
mQTL database [48]	1000 mother–child pairs across the life course; based on blood	http://mqtldb.org
BIOS QTL browser	3841 adult blood samples of varying ages	https://genenetwork.nl/biosqtlbrowser
GoDMC [49]	Largest mQTL consortium to date; focus on blood tissue	http://www.godmc.org.uk/projects.html
Brain xQTL Serve [49]	411 frontal cortex brain samples of older adults	http://mostafavilab.stat.ubc.ca/xqtl
Brain Epigenomics [50]	166 foetal brain samples	https://epigenetics.essex.ac.uk/mQTL

**Table 4:** Resources providing genome-wide association study (GWAS) summary statistics for (mental health) traits. EMBL-EBI: European Molecular Biology Laboratory - European Bioinformatics Institute; NHGRI: National Human Genome Research Institute; ENIGMA: Enhancing Neuro Imaging Genetics Through Meta Analysis.

Resource	Description	Link
MRInstruments	R package that contains a number of data files from various sources to provide instruments in two-sample MR	https://github.com/MRCIEU/MRInstruments
Phenoscanner [56]	Lists over 65 billion GWAS associations, hosted at the University of Cambridge	http://www.phenoscanner.medschl.cam.ac.uk
GWAS catalogue [57]	Curated catalogue in collaborative between the EMBL-EBI and NHGRI	https://www.ebi.ac.uk/gwas
Psychiatric Genomics Consortium [58]	Genome-wide summary data for psychiatric disorders	https://www.med.unc.edu/pgc/results-and-downloads
ENIGMA brain structure [59]	Genome-wide summary data for brain structure phenotypes	http://enigma.ini.usc.edu/research/download-enigma-gwas-results

**Table 5:** Selection of resources to aid in the biological characterisation of DNA methylation findings.

Resource	Description	Link
ENCODE data [72]	Tissue-specific regulatory elements across a wide range of tissues	www.encodeproject.org; or via www.ucsc.genome.edu
Roadmap Epigenetics Project [84]	Tissue-specific regulatory elements, specifically in brain tissue	http://www.roadmapepigenomics.org; or via www.ucsc.genome.edu
PsychENCODE [75]	Brain-specific tissue and single-cell transcriptomic and epigenomic data	http://www.psychencode.org/
EWAS catalogue	Manually curated and quality controlled catalogue of epigenome-wide association studies	ewascatalog.org
Imprinted genes	List of imprinted genes (by species)	http://www.geneimprint.com/site/genes-by-species

**Table 6:** Resources for cross-tissue comparisons of methylation signals.

Resource	Description	Link
BECon [86]	Cross-tissue correlations of 450k probes across paired blood and brain regions of 16 individuals	https://redgar598.shinyapps.io/BECon
Brain Epigenomics	Cross-tissue correlations of 450k probes across paired blood and brain regions of 74 individuals	https://epigenetics.essex.ac.uk/bloodbrain
MethBase [87]	Methylation profiles across tissues, development and species, based on bisulphite sequencing	http://smithlabresearch.org/software/methbase
eFORGE [88]	Analysis of cell type-specific signals in epigenomic data	http://eforge.cs.ucl.ac.uk/

**Table 7:** Resources to investigate tissue-specific gene expression. eQTL: expression quantitative trait locus.

Resource	Description	Link
Human Protein Atlas	Expression profiles for all protein-coding genes in 44 tissues and organs in the human body	https://www.proteinatlas.org/humanproteome
Genotype-Tissue Expression project (GTEx)	Information on tissue-specific gene expression, regulation and eQTL information, based on 53 non-diseased tissues across 714 individuals	https://gtexportal.org/home/
BIOS QTL Browser [49]	Methylation QTL data, based on up to 3,841 whole-blood samples	https://genenetwork.nl/biosqtlbrowser/
EMBL-EPI expression atlas	Gene expression profiles across development, based on a range of resources	https://www.ebi.ac.uk/gxa/home
Human Brain Atlas [91]	Multimodal atlas of the human brain, integrating highly detailed anatomic and genomic information based on six adult brains	http://human.brain-map.org/
Developing Human [92] Brain	Human transcriptome in up to 16 brain regions from 4 weeks post conception to over 40 years	http://www.brainspan.org/
BrainCloud [93]	Gene expression and their genetic control in the dorsolateral prefrontal cortex of normal subjects across the lifespan	http://braincloud.jhmi.edu/
PsychENCODE [75]	Integration of expression and other regulatory elements across different brain cell types, regions and developmental periods	http://www.psychencode.org/

## References

[1] Montano C., Taub M.A., Jaffe A., Briem E., Feinberg J.I., Trygvadottir R., Idrizi A., Runarsson A., Berndsen B., Gur R.C. (2016). Association of DNA Methylation Differences With Schizophrenia in an Epigenome-Wide Association Study. JAMA Psychiatry.

[2] Bani-Fatemi A., Jeremian R., Wang K.Z., Silveira J., Zai C., Kolla N.J., Graff A., Gerretsen P., Strauss J., De Luca V. (2018). Epigenome-wide association study of suicide attempt in schizophrenia. J. Psychiatr. Res..

[3] Story Jovanova O., Nedeljkovic I., Spieler D., Walker R.M., Liu C., Luciano M., Bressler J., Brody J., Drake A.J., Evans K.L. (2018). DNA Methylation Signatures of Depressive Symptoms in Middle-aged and Elderly Persons: Meta-analysis of Multiethnic Epigenome-wide Studies. JAMA Psychiatry.

[4] Baselmans B.M., van Dongen J., Nivard M.G., Lin B.D., Consortium B., Zilhao N.R., Boomsma D.I., Bartels M. (2015). Epigenome-Wide Association Study of Wellbeing. Twin Res. Hum. Genet..

[5] Shimada-Sugimoto M., Otowa T., Miyagawa T., Umekage T., Kawamura Y., Bundo M., Iwamoto K., Tochigi M., Kasai K., Kaiya H. (2017). Epigenome-wide association study of DNA methylation in panic disorder. Clin. Epigenetics.

[6] Shimada M., Otowa T., Miyagawa T., Umekage T., Kawamura Y., Bundo M., Iwamoto K., Ikegame T., Tochigi M., Kasai K. (2018). An epigenome-wide methylation study of healthy individuals with or without depressive symptoms. J. Hum. Genet..

[7] Kuan P.F., Waszczuk M.A., Kotov R., Marsit C.J., Guffanti G., Gonzalez A., Yang X., Koenen K., Bromet E., Luft B.J. (2017). An epigenome-wide DNA methylation study of PTSD and depression in World Trade Center responders. Transl. Psychiatry.

[8] Marioni R.E., McRae A.F., Bressler J., Colicino E., Hannon E., Li S., Prada D., Smith J.A., Trevisi L., Tsai P.C. (2018). Meta-analysis of epigenome-wide association studies of cognitive abilities. Mol. Psychiatry.

[9] Walton E., Pingault J.B., Cecil C.A., Gaunt T.R., Relton C.L., Mill J., Barker E.D. (2017). Epigenetic profiling of ADHD symptoms trajectories: A prospective, methylome-wide study. Mol. Psychiatry.

[10] Barker E.D., Walton E., Cecil C.A.M., Rowe R., Jaffee S.R., Maughan B., O’Connor T.G., Stringaris A., Meehan A.J., McArdle W. (2018). A Methylome-Wide Association Study of Trajectories of Oppositional Defiant Behaviors and Biological Overlap with Attention Deficit Hyperactivity Disorder. Child Dev..

[11] De Vocht F., Suderman M., Tilling K., Heron J., Howe L.D., Campbell R., Hickman M., Relton C. (2018). DNA methylation from birth to late adolescence and development of multiple-risk behaviours. J. Affect. Disord..

[12] Cecil C.A., Walton E., Smith R.G., Viding E., McCrory E.J., Relton C.L., Suderman M., Pingault J.B., McArdle W., Gaunt T.R. (2016). DNA methylation and substance-use risk: A prospective, genome-wide study spanning gestation to adolescence. Transl. Psychiatry.

[13] Cecil C.A.M., Walton E., Jaffee S.R., O’Connor T., Maughan B., Relton C.L., Smith R.G., McArdle W., Gaunt T.R., Ouellet-Morin I. (2018). Neonatal DNA methylation and early-onset conduct problems: A genome-wide, prospective study. Dev. Psychopathol..

[14] Cecil C.A.M., Walton E., Pingault J.B., Provencal N., Pappa I., Vitaro F., Cote S., Szyf M., Tremblay R.E., Tiemeier H. (2018). DRD4 methylation as a potential biomarker for physical aggression: An epigenome-wide, cross-tissue investigation. Am. J. Med. Genet. B Neuropsychiatr. Genet..

[15] Mitjans M., Seidel J., Begemann M., Bockhop F., Moya-Higueras J., Bansal V., Wesolowski J., Seelbach A., Ibanez M.I., Kovacevic F. (2018). Violent aggression predicted by multiple pre-adult environmental hits. Mol. Psychiatry.

[16] Andrews S.V., Sheppard B., Windham G.C., Schieve L.A., Schendel D.E., Croen L.A., Chopra P., Alisch R.S., Newschaffer C.J., Warren S.T. (2018). Case-control meta-analysis of blood DNA methylation and autism spectrum disorder. Mol. Autism..

[17] Hannon E., Schendel D., Ladd-Acosta C., Grove J., Hansen C.S., Andrews S.V., Hougaard D.M., Bresnahan M., Mors O. (2018). Elevated polygenic burden for autism is associated with differential DNA methylation at birth. Genome Med..

[18] Long H.Y., Feng L., Kang J., Luo Z.H., Xiao W.B., Long L.L., Yan X.X., Zhou L., Xiao B. (2017). Blood DNA methylation pattern is altered in mesial temporal lobe epilepsy. Sci. Rep..

[19] Shimada M., Miyagawa T., Toyoda H., Tokunaga K., Honda M. (2018). Epigenome-wide association study of DNA methylation in narcolepsy: An integrated genetic and epigenetic approach. Sleep.

[20] Kaut O., Schmitt I., Tost J., Busato F., Liu Y., Hofmann P., Witt S.H., Rietschel M., Frohlich H., Wullner U. (2017). Epigenome-wide DNA methylation analysis in siblings and monozygotic twins discordant for sporadic Parkinson’s disease revealed different epigenetic patterns in peripheral blood mononuclear cells. Neurogenetics.

[21] Karlsson Linner R., Marioni R.E., Rietveld C.A., Simpkin A.J., Davies N.M., Watanabe K., Armstrong N.J., Auro K., Baumbach C., Bonder M.J. (2017). An epigenome-wide association study meta-analysis of educational attainment. Mol. Psychiatry.

[22] Crawford B., Craig Z., Mansell G., White I., Smith A., Spaull S., Imm J., Hannon E., Wood A., Yaghootkar H. (2018). DNA methylation and inflammation marker profiles associated with a history of depression. Hum. Mol. Genet..

[23] Bakulski K.M., Halladay A., Hu V.W., Mill J., Fallin M.D. (2016). Epigenetic Research in Neuropsychiatric Disorders: The “Tissue Issue”. Curr. Behav. Neurosci. Rep..

[24] Ladd-Acosta C., Hansen K.D., Briem E., Fallin M.D., Kaufmann W.E., Feinberg A.P. (2014). Common DNA methylation alterations in multiple brain regions in autism. Mol. Psychiatry.

[25] Kinoshita M., Numata S., Tajima A., Yamamori H., Yasuda Y., Fujimoto M., Watanabe S., Umehara H., Shimodera S., Nakazawa T. (2017). Effect of Clozapine on DNA Methylation in Peripheral Leukocytes from Patients with Treatment-Resistant Schizophrenia. Int. J. Mol. Sci..

[26] Hutnick L.K., Golshani P., Namihira M., Xue Z., Matynia A., Yang X.W., Silva A.J., Schweizer F.E., Fan G. (2009). DNA hypomethylation restricted to the murine forebrain induces cortical degeneration and impairs postnatal neuronal maturation. Hum. Mol. Genet..

[27] Feng J., Zhou Y., Campbell S.L., Le T., Li E., Sweatt J.D., Silva A.J., Fan G. (2010). Dnmt1 and Dnmt3a maintain DNA methylation and regulate synaptic function in adult forebrain neurons. Nat. Neurosci..

[28] Morris M.J., Na E.S., Autry A.E., Monteggia L.M. (2016). Impact of DNMT1 and DNMT3a forebrain knockout on depressive- and anxiety like behavior in mice. Neurobiol. Learn. Mem..

[29] Liu X.S., Wu H., Krzisch M., Wu X., Graef J., Muffat J., Hnisz D., Li C.H., Yuan B., Xu C. (2018). Rescue of Fragile X Syndrome Neurons by DNA Methylation Editing of the FMR1 Gene. Cell.

[30] Felix J.F., Joubert B.R., Baccarelli A.A., Sharp G.C., Almqvist C., Annesi-Maesano I., Arshad H., Baiz N., Bakermans-Kranenburg M.J., Bakulski K.M. (2017). Cohort Profile: Pregnancy And Childhood Epigenetics (PACE) Consortium. Int. J. Epidemiol..

[31] Psaty B.M., O’Donnell C.J., Gudnason V., Lunetta K.L., Folsom A.R., Rotter J.I., Uitterlinden A.G., Harris T.B., Witteman J.C., Boerwinkle E. (2009). Cohorts for Heart and Aging Research in Genomic Epidemiology (CHARGE) Consortium: Design of prospective meta-analyses of genome-wide association studies from 5 cohorts. Circ. Cardiovasc. Genet..

[32] Ligthart S., Marzi C., Aslibekyan S., Mendelson M.M., Conneely K.N., Tanaka T., Colicino E., Waite L.L., Joehanes R., Guan W. (2016). DNA methylation signatures of chronic low-grade inflammation are associated with complex diseases. Genome Biol..

[33] Min J.L., Hemani G., Davey Smith G., Relton C., Suderman M. (2018). Meffil: Efficient normalization and analysis of very large DNA methylation datasets. Bioinformatics.

[34] Barrett T., Troup D.B., Wilhite S.E., Ledoux P., Evangelista C., Kim I.F., Tomashevsky M., Marshall K.A., Phillippy K.H., Sherman P.M. (2011). NCBI GEO: Archive for functional genomics data sets—10 years on. Nucleic Acids Res..

[35] Li H., Chen R., Cai J., Cui X., Huang N., Kan H. (2018). Short-term exposure to fine particulate air pollution and genome-wide DNA methylation: A randomized, double-blind, crossover trial. Environ. Int..

[36] Richmond R.C., Sharp G.C., Herbert G., Atkinson C., Taylor C., Bhattacharya S., Campbell D., Hall M., Kazmi N., Gaunt T. (2018). The long-term impact of folic acid in pregnancy on offspring DNA methylation: Follow-up of the Aberdeen Folic Acid Supplementation Trial (AFAST). Int. J. Epidemiol..

[37] Tobi E.W., Goeman J.J., Monajemi R., Gu H., Putter H., Zhang Y., Slieker R.C., Stok A.P., Thijssen P.E., Muller F. (2014). DNA methylation signatures link prenatal famine exposure to growth and metabolism. Nat. Commun..

[38] Van den Broek T., Fleischmann M. (2017). Prenatal famine exposure and mental health in later midlife. Aging Ment. Health.

[39] Cao-Lei L., Elgbeili G., Massart R., Laplante D.P., Szyf M., King S. (2015). Pregnant women’s cognitive appraisal of a natural disaster affects DNA methylation in their children 13 years later: Project Ice Storm. Transl. Psychiatry.

[40] Walder D.J., Laplante D.P., Sousa-Pires A., Veru F., Brunet A., King S. (2014). Prenatal maternal stress predicts autism traits in 6(1/2) year-old children: Project Ice Storm. Psychiatry Res..

[41] Davey Smith G., Hemani G. (2014). Mendelian randomization: Genetic anchors for causal inference in epidemiological studies. Hum. Mol. Genet..

[42] Zheng J., Baird D., Borges M.C., Bowden J., Hemani G., Haycock P., Evans D.M., Smith G.D. (2017). Recent Developments in Mendelian Randomization Studies. Curr. Epidemiol. Rep..

[43] Pingault J.B., O’Reilly P.F., Schoeler T., Ploubidis G.B., Rijsdijk F., Dudbridge F. (2018). Using genetic data to strengthen causal inference in observational research. Nat. Rev. Genet..

[44] Teumer A. (2018). Common Methods for Performing Mendelian Randomization. Front. Cardiovasc. Med..

[45] Richardson T.G., Haycock P.C., Zheng J., Timpson N.J., Gaunt T.R., Smith G.D., Relton C.L., Hemani G. (2017). Systematic Mendelian randomization framework elucidates hundreds of genetic loci which may influence disease through changes in DNA methylation levels. bioRxiv.

[46] Walton E., Hemani G., Dehghan A., Relton C., Smith G.D. (2018). Systematic evaluation of the causal relationship between DNA methylation and C-reactive protein. bioRxiv.

[47] Lin D., Chen J., Perrone-Bizzozero N., Bustillo J.R., Du Y., Calhoun V.D., Liu J. (2018). Characterization of cross-tissue genetic-epigenetic effects and their patterns in schizophrenia. Genome Med..

[48] Gaunt T.R., Shihab H.A., Hemani G., Min J.L., Woodward G., Lyttleton O., Zheng J., Duggirala A., McArdle W.L., Ho K. (2016). Systematic identification of genetic influences on methylation across the human life course. Genome Biol..

[49] Bonder M.J., Luijk R., Zhernakova D.V., Moed M., Deelen P., Vermaat M., van Iterson M., van Dijk F., van Galen M., Bot J. (2017). Disease variants alter transcription factor levels and methylation of their binding sites. Nat. Genet..

[50] Hannon E., Spiers H., Viana J., Pidsley R., Burrage J., Murphy T.M., Troakes C., Turecki G., O’Donovan M.C., Schalkwyk L.C. (2016). Methylation QTLs in the developing brain and their enrichment in schizophrenia risk loci. Nat. Neurosci..

[51] Chausse P. (2010). Computing Generalized Method of Moments and Generalized Empirical Likelihood with R. J. Stat. Softw..

[52] Thomas D.C., Lawlor D.A., Thompson J.R. (2007). Re: Estimation of bias in nongenetic observational studies using “Mendelian triangulation” by Bautista et al. Ann. Epidemiol..

[53] Hemani G., Zheng J., Elsworth B., Wade K., Haberland V., Baird D., Laurin C., Burgess S., Bowden J., Langdon R. (2018). The MR-base platform enables systematic causal inference across the phenome. eLife.

[54] Yavorska O.O., Burgess S. (2017). MendelianRandomization: An R package for performing Mendelian randomization analyses using summarized data. Int. J. Epidemiol..

[55] Bowden J., Davey Smith G., Burgess S. (2015). Mendelian randomization with invalid instruments: Effect estimation and bias detection through Egger regression. Int. J. Epidemiol..

[56] Staley J.R., Blackshaw J., Kamat M.A., Ellis S., Surendran P., Sun B.B., Paul D.S., Freitag D., Burgess S., Danesh J. (2016). PhenoScanner: A database of human genotype-phenotype associations. Bioinformatics.

[57] MacArthur J., Bowler E., Cerezo M., Gil L., Hall P., Hastings E., Junkins H., McMahon A., Milano A., Morales J. (2017). The new NHGRI-EBI Catalog of published genome-wide association studies (GWAS Catalog). Nucleic Acids Res..

[58] Sullivan P.F., Agrawal A., Bulik C.M., Andreassen O.A., Borglum A.D., Breen G., Cichon S., Edenberg H.J., Faraone S.V., Gelernter J. (2018). Psychiatric Genomics: An Update and an Agenda. Am. J. Psychiatry.

[59] Thompson P.M., Andreassen O.A., Arias-Vasquez A., Bearden C.E., Boedhoe P.S., Brouwer R.M., Buckner R.L., Buitelaar J.K., Bulayeva K.B., Cannon D.M. (2017). ENIGMA and the individual: Predicting factors that affect the brain in 35 countries worldwide. Neuroimage.

[60] Kirkbride J.B., Susser E., Kundakovic M., Kresovich J.K., Davey Smith G., Relton C.L. (2012). Prenatal nutrition, epigenetics and schizophrenia risk: Can we test causal effects?. Epigenomics.

[61] Caramaschi D., Sharp G.C., Nohr E.A., Berryman K., Lewis S.J., Davey Smith G., Relton C.L. (2017). Exploring a causal role of DNA methylation in the relationship between maternal vitamin B12 during pregnancy and child’s IQ at age 8, cognitive performance and educational attainment: A two-step Mendelian randomization study. Hum. Mol. Genet..

[62] Viana J., Hannon E., Dempster E., Pidsley R., Macdonald R., Knox O., Spiers H., Troakes C., Al-Saraj S., Turecki G. (2017). Schizophrenia-associated methylomic variation: Molecular signatures of disease and polygenic risk burden across multiple brain regions. Hum. Mol. Genet..

[63] Hannon E., Dempster E., Viana J., Burrage J., Smith A.R., Macdonald R., St Clair D., Mustard C., Breen G., Therman S. (2016). An integrated genetic-epigenetic analysis of schizophrenia: Evidence for co-localization of genetic associations and differential DNA methylation. Genome Biol..

[64] Choi S.W., Mak T.S.H., O’Reilly P. (2018). A guide to performing Polygenic Risk Score analyses. bioRxiv.

[65] Giambartolomei C., Vukcevic D., Schadt E.E., Franke L., Hingorani A.D., Wallace C., Plagnol V. (2014). Bayesian test for colocalisation between pairs of genetic association studies using summary statistics. PLoS Genet..

[66] Walton E., Hass J., Liu J., Roffman J.L., Bernardoni F., Roessner V., Kirsch M., Schackert G., Calhoun V., Ehrlich S. (2016). Correspondence of DNA Methylation Between Blood and Brain Tissue and Its Application to Schizophrenia Research. Schizophr. Bull..

[67] Mohammad-Zadeh L.F., Moses L., Gwaltney-Brant S.M. (2008). Serotonin: A review. J. Vet. Pharmacol. Ther..

[68] Biomarkers Definitions Working G. (2001). Biomarkers and surrogate endpoints: Preferred definitions and conceptual framework. Clin. Pharmacol. Ther..

[69] Mitchell R.E., Paternoster L., Davey Smith G. (2018). Mendelian Randomization in Case Only Studies: A Promising Approach to be Applied With Caution. Am. J. Cardiol..

[70] Ng B., White C.C., Klein H.U., Sieberts S.K., McCabe C., Patrick E., Xu J., Yu L., Gaiteri C., Bennett D.A. (2017). An xQTL map integrates the genetic architecture of the human brain’s transcriptome and epigenome. Nat. Neurosci..

[71] Severin J., Waterhouse A.M., Kawaji H., Lassmann T., van Nimwegen E., Balwierz P.J., de Hoon M.J., Hume D.A., Carninci P., Hayashizaki Y. (2009). FANTOM4 EdgeExpressDB: An integrated database of promoters, genes, microRNAs, expression dynamics and regulatory interactions. Genome Biol..

[72] Encode Project Consortium (2012). An integrated encyclopedia of DNA elements in the human genome. Nature.

[73] Li M., Santpere G., Imamura Kawasawa Y., Evgrafov O.V., Gulden F.O., Pochareddy S., Sunkin S.M., Li Z., Shin Y., Zhu Y. (2018). Integrative functional genomic analysis of human brain development and neuropsychiatric risks. Science.

[74] Gandal M.J., Zhang P., Hadjimichael E., Walker R.L., Chen C., Liu S., Won H., van Bakel H., Varghese M., Wang Y. (2018). Transcriptome-wide isoform-level dysregulation in ASD, schizophrenia, and bipolar disorder. Science.

[75] Wang D., Liu S., Warrell J., Won H., Shi X., Navarro F.C.P., Clarke D., Gu M., Emani P., Yang Y.T. (2018). Comprehensive functional genomic resource and integrative model for the human brain. Science.

[76] Zhu Y., Sousa A.M.M., Gao T., Skarica M., Li M., Santpere G., Esteller-Cucala P., Juan D., Ferrández-Peral L., Gulden F.O. (2018). Spatiotemporal transcriptomic divergence across human and macaque brain development. Science.

[77] Amiri A., Coppola G., Scuderi S., Wu F., Roychowdhury T., Liu F., Pochareddy S., Shin Y., Safi A., Song L. (2018). Transcriptome and epigenome landscape of human cortical development modeled in organoids. Science.

[78] Rajarajan P., Borrman T., Liao W., Schrode N., Flaherty E., Casiño C., Powell S., Yashaswini C., LaMarca E.A., Kassim B. (2018). Neuron-specific signatures in the chromosomal connectome associated with schizophrenia risk. Science.

[79] An J.-Y., Lin K., Zhu L., Werling D.M., Dong S., Brand H., Wang H.Z., Zhao X., Schwartz G.B., Collins R.L. (2018). Genome-wide de novo risk score implicates promoter variation in autism spectrum disorder. Science.

[80] Rhie S.K., Schreiner S., Witt H., Armoskus C., Lay F.D., Camarena A., Spitsyna V.N., Guo Y., Berman B.P., Evgrafov O.V. (2018). Using 3D epigenomic maps of primary olfactory neuronal cells from living individuals to understand gene regulation. Science Advances.

[81] Kozlenkov A., Li J., Apontes P., Hurd Y.L., Byne W.M., Koonin E.V., Wegner M., Mukamel E.A., Dracheva S. (2018). A unique role for DNA (hydroxy)methylation in epigenetic regulation of human inhibitory neurons. Sci. Adv..

[82] Chen C., Meng Q., Xia Y., Ding C., Wang L., Dai R., Cheng L., Gunaratne P., Gibbs R.A., Min S. (2018). The transcription factor POU3F2 regulates a gene coexpression network in brain tissue from patients with psychiatric disorders. Sci. Transl. Med..

[83] Meng Q., Wang K., Brunetti T., Xia Y., Jiao C., Dai R., Fitzgerald D., Thomas A., Jay L., Eckart H. (2018). The *DGCR5* long noncoding RNA may regulate expression of several schizophrenia-related genes. Sci. Transl. Med..

[84] Roadmap Epigenomics C., Kundaje A., Meuleman W., Ernst J., Bilenky M., Yen A., Heravi-Moussavi A., Kheradpour P., Zhang Z., Wang J. (2015). Integrative analysis of 111 reference human epigenomes. Nature.

[85] Van Baak T.E., Coarfa C., Dugue P.A., Fiorito G., Laritsky E., Baker M.S., Kessler N.J., Dong J., Duryea J.D., Silver M.J. (2018). Epigenetic supersimilarity of monozygotic twin pairs. Genome Biol..

[86] Edgar R.D., Jones M.J., Meaney M.J., Turecki G., Kobor M.S. (2017). BECon: A tool for interpreting DNA methylation findings from blood in the context of brain. Transl. Psychiatry.

[87] Song Q., Decato B., Hong E.E., Zhou M., Fang F., Qu J., Garvin T., Kessler M., Zhou J., Smith A.D. (2013). A reference methylome database and analysis pipeline to facilitate integrative and comparative epigenomics. PLoS ONE.

[88] Breeze C.E., Paul D.S., van Dongen J., Butcher L.M., Ambrose J.C., Barrett J.E., Lowe R., Rakyan V.K., Iotchkova V., Frontini M. (2016). eFORGE: A Tool for Identifying Cell Type-Specific Signal in Epigenomic Data. Cell Rep..

[89] Schlosberg C.E., VanderKraats N.D., Edwards J.R. (2017). Modeling complex patterns of differential DNA methylation that associate with gene expression changes. Nucleic Acids Res..

[90] Keil J.M., Qalieh A., Kwan K.Y. (2018). Brain transcriptome databases: A user’s guide. J. Neurosci..

[91] Hawrylycz M.J., Lein E.S., Guillozet-Bongaarts A.L., Shen E.H., Ng L., Miller J.A., van de Lagemaat L.N., Smith K.A., Ebbert A., Riley Z.L. (2012). An anatomically comprehensive atlas of the adult human brain transcriptome. Nature.

[92] Miller J.A., Ding S.L., Sunkin S.M., Smith K.A., Ng L., Szafer A., Ebbert A., Riley Z.L., Royall J.J., Aiona K. (2014). Transcriptional landscape of the prenatal human brain. Nature.

[93] Colantuoni C., Lipska B.K., Ye T., Hyde T.M., Tao R., Leek J.T., Colantuoni E.A., Elkahloun A.G., Herman M.M., Weinberger D.R. (2011). Temporal dynamics and genetic control of transcription in the human prefrontal cortex. Nature.

[94] Munafò M.R., Yalcin B., Willis-Owen S.A., Flint J. (2008). Association of the dopamine D4 receptor (DRD4) gene and approach-related personality traits: Meta-analysis and new data. Biol. Psychiatry.

[95] Ptácek R., Kuzelová H., Stefano G.B. (2011). Dopamine D4 receptor gene DRD4 and its association with psychiatric disorders. Med. Sci. Monit..

[96] Di Ciano P., Grandy D.K., Le Foll B. (2014). Dopamine D4 receptors in psychostimulant addiction. Adv. Pharmacol..

[97] Lindsley C.W., Hopkins C.R. (2017). Return of D4 Dopamine Receptor Antagonists in Drug Discovery. J. Med. Chem..

[98] Cirillo E., Parnell L.D., Evelo C.T. (2017). A Review of Pathway-Based Analysis Tools That Visualize Genetic Variants. Front. Genet..

[99] Kao P.Y.P., Leung K.H., Chan L.W.C., Yip S.P., Yap M.K.H. (2017). Pathway analysis of complex diseases for GWAS, extending to consider rare variants, multi-omics and interactions. Biochim. Biophys. Acta (BBA) Gen. Subj..

[100] Watanabe K., Taskesen E., van Bochoven A., Posthuma D. (2017). Functional mapping and annotation of genetic associations with FUMA. Nat. Commun..

[101] Aberg K.A., Dean B., Shabalin A.A., Chan R.F., Han L.K.M., Zhao M., van Grootheest G., Xie L.Y., Milaneschi Y., Clark S.L. (2018). Methylome-wide association findings for major depressive disorder overlap in blood and brain and replicate in independent brain samples. Mol. Psychiatry.

[102] Jang H.S., Shin W.J., Lee J.E., Do J.T. (2017). CpG and Non-CpG Methylation in Epigenetic Gene Regulation and Brain Function. Genes (Basel).

[103] Rizzardi L.F., Hickey P.F., Rodriguez DiBlasi V., Tryggvadottir R., Callahan C.M., Idrizi A., Hansen K.D., Feinberg A.P. (2019). Neuronal brain-region-specific DNA methylation and chromatin accessibility are associated with neuropsychiatric trait heritability. Nat. Neurosci..

